# Estradiol negatively associates with metabolic dysfunction-associated steatotic liver disease in children

**DOI:** 10.3389/fendo.2026.1840658

**Published:** 2026-07-06

**Authors:** Judith Lubrecht, Robert Kleemann, Bjorn Winkens, Ger Koek, Annemieke Heijboer, Anita Vreugdenhil

**Affiliations:** 1 Centre for Overweight Adolescent and Children’s Healthcare (COACH), Maastricht University Medical Center+, MosaKids Children’s Hospital, Maastricht, Netherlands; 2 Department of Pediatrics, Maastricht University Medical Center+, MosaKids Children’s Hospital, Maastricht, Netherlands; 3 School of Nutrition and Translational Research (NUTRIM), Maastricht University, Maastricht, Netherlands; 4 Department of Metabolic Health Research, The Netherlands Organization for Applied Scientific Research (TNO), Leiden, Netherlands; 5 Department of Methodology and Statistics, Care and Public Health Research Institute (CAPHRI), Maastricht University, Maastricht, Netherlands; 6 Department of Gastroenterology, Maastricht University Medical Center+, Maastricht, Netherlands; 7 Endocrine Laboratory, Department of Laboratory Medicine, Amsterdam UMC, location University of Amsterdam, Amsterdam, Netherlands; 8 Endocrine Laboratory, Department of Laboratory Medicine, Amsterdam UMC, location Vrije Universiteit Amsterdam, Amsterdam, Netherlands; 9 Amsterdam Gastroenterology, Endocrinology & Metabolism, Amsterdam, Netherlands

**Keywords:** adolescent, child, estradiol, fatty liver, MASLD, NAFLD, obesity, sex differences

## Abstract

**Background:**

Metabolic dysfunction-associated steatotic liver disease (MASLD) is the leading cause of chronic liver disease in children and adolescents. Studies in adults indicate that MASLD is more prevalent in males compared to females and that sex hormones may play a significant role in MASLD development. However, limited pediatric research is available on the relation between sex hormones and MASLD. This study investigates associations between sex hormones and MASLD in children with overweight and obesity.

**Methods:**

A cross-sectional study was conducted in children with overweight and obesity in a tertiary care center (Maastricht UMC+). A comprehensive serum sex hormone panel, serum alanine aminotransferase (ALT) and hepatic steatosis on ultrasound (HS) were determined in 290 participants. Controlled Attenuation Parameter (CAP, measured with FibroScan) was available in 75 participants. Associations between sex hormones and MASLD parameters (ALT, HS and CAP) were investigated.

**Results:**

This study found that estradiol, Anti-Müllerian hormone (AMH) and sex hormone-binding globulin (SHBG) inversely associated with ALT (p=0.018, p=0.048 and p<0.001). Free (FT) and bioavailable (BT) testosterone and dehydroepiandrosterone sulfate (DHEAS) positively associated with ALT (both p=0.006). In addition to this, AMH and SHBG inversely associated with HS (p=0.028 and p<0.001), while FT, BioT and DHEAS positively associated with HS (p=0.024, p=0.024 and p=0.042). Lastly, AMH and SHBG inversely associated with CAP (p=0.04 and p<0.001), whereas follicle stimulating hormone, total testosterone (TT), FT, BioT, androstenedione and DHEAS positively associated (p=0.034, p=0.04, p=0.032, p=0.032, p=0.007 and p=0.003).

**Conclusions:**

Estradiol, AMH and SHGB inversely associate with MASLD parameters, while androgens, including TT, FT, BioT, and DHEAS, positively associate with MASLD parameters in children with overweight and obesity.

## Introduction

Metabolic dysfunction-associated steatotic liver disease (MASLD) has become the leading chronic liver disease in children and adolescents, with studies reporting a pooled prevalence of 34.2% in children with obesity (1). MASLD encompasses several different liver disorders, ranging from simple steatosis and non-alcoholic steatohepatitis, to liver fibrosis and cirrhosis, culminating in end-stage liver disease (2). MASLD is associated with different intra- and extrahepatic comorbidities, including type II diabetes and cardiovascular disease (2, 3). Moreover, MASLD is one of the leading indications for liver transplants in adults (2).

The prevalence of MASLD appears to be dependent on both age and sex (4). It is significantly higher in men compared to women of reproductive age (5, 6). However, prevalence steeply increases in women after menopause and eventually surpasses prevalence observed in men in the same age category (4, 6). Differences in the prevalence of MASLD between boys and girls have also been reported, with increasing prevalence in boys through puberty compared to girls (7). These results suggest that sex hormones may play an important role in MASLD. Preclinical and clinical adult studies that examined possible associations between MASLD and hormone parameters support these epidemiological findings, reporting that estrogens are inversely associated with MASLD in females, while elevated levels of androgens perpetuate MASLD development in females (8, 9). Studies on androgen signaling in males report conflicting results, with some studies indicating that androgens protect against MASLD, while other population based studies find positive associations between circulating levels of testosterone and hepatic steatosis in men (8, 10). A single study has been performed in children with MASLD in the United States (11). The authors found that testosterone inversely associated with steatosis and fibrosis in boys, while testosterone was associated with increasing steatosis severity in girls. In the same study, SHBG was inversely associated with steatosis severity in boys and girls and with portal inflammation in girls. Higher concentrations of estrone, estradiol and testosterone were associated with lower portal inflammation grade in both boys and girls (11). However, these results may not be applicable to other populations, as studies have shown that sex hormone concentrations are subject to geographical and ethnic variation. In addition to this, Mueller et al. measured sex hormones with immunoassays that yield inferior results compared to measurements using mass spectrometry, i.e. the gold standard methodology. Our study investigates the associations between a comprehensive set of sex hormones, of which several are measured with mass spectrometry, and MASLD in a European cohort of children with overweight, obesity and severe obesity.

## Materials and methods

### Setting

This cross-sectional study was designed and conducted within the Centre for Overweight Adolescent and Children’s Healthcare (COACH), at the Maastricht University Medical Center + (MUMC+) in the Netherlands. COACH is a tertiary care center that offers ongoing outpatient combined lifestyle intervention (CLI) to children with overweight and obesity and their families, aimed at building a healthier lifestyle and healthy weight. Children are referred to COACH by general practitioners, youth health services, pediatricians, or other healthcare providers, according to their standard of care guidelines.

A comprehensive health assessment is conducted before children start CLI to assess possible causes, comorbidities and weight-related health risks. Based on this health assessment, a personalized care plan is tailored to the needs and wishes of the child and family. The basis of this personalized care plan is lifestyle coaching, provided in individual outpatient clinic visits every 4–6 weeks. Topics include but are not limited to physical activity, nutrition, eating habits, sleep habits, psychological wellbeing, and social aspects of healthy living. Additional support from other healthcare providers is employed when deemed necessary. Besides individual counselling, voluntary group activities related to nutrition and physical activity are offered to children and their families. Comprehensive details of this intervention are described elsewhere (12).

### Study population

Participants were recruited from COACH. Children, aged 4–18 years, eligible for participation had overweight, obesity or severe obesity at baseline according to the International Obesity Task Force (IOTF) criteria (13). They were excluded if data on serum ALT concentrations, any hormone parameters, abdominal ultrasound or Tanner stages were missing, if they were diagnosed with other causes for ALT elevation than hepatic steatosis, if they were diagnosed with aberrant puberty development, or if they used hepatotoxic or hormone medication including anti-epileptics, anti-psychotics, leukotriene receptor antagonists or hormonal contraceptives. A total of 290 children with overweight and obesity qualified for inclusion in this study.

This study was conducted according to the guidelines of the Declaration of Helsinki and STROBE guidelines and was approved by the Medical Ethical Committee of the Maastricht University Medical Centre (MEC 13-4-130; clinicaltrial.gov (NCT02091544)). Written informed consent from all necessary parties (both parents or legal guardians, and the child if aged ≥12 years) was obtained before inclusion.

### Anthropometric measurements

Anthropometric measurements were performed with children barefoot and wearing underwear only. Body weight was measured using a digital, calibrated scale (Seca, Chino, CA, USA). Height was measured using a rigid wall-mounted stadiometer (De Grood Metaaltechniek, Nijmegen, the Netherlands). Body mass index (BMI) was calculated and BMI z-scores were obtained using the Growth Analyzer embedded in the electronic patient file (Growth Analyzer VE, Rotterdam, the Netherlands). All children were classified as having overweight, obesity, or severe obesity, according to IOTF criteria (13).

Sex was determined as biological sex assigned at birth. Puberty stage was determined during physical examination, using the Tanner stages (14, 15). Children were divided in three subgroups according to their genital (G) or mammae (M) Tanner stage: prepubertal (Tanner G/M-stage 1), intrapubertal (Tanner G/M-stage 2-3) or postpubertal (Tanner G/M-stage 4-5) (16, 17). Stages 4 and 5 were combined to balance groups. Tanner stages were complete and objectively verified for all 290 children.

### Laboratory measurements

Venous blood sampling was performed in a fasted state. Alanine aminotransferase (ALT) was determined with enzymatic spectrophotometric assays (Cobas 8000 instrument, Roche Diagnostics, Mannheim, Germany). Luteinizing hormone (LH), follicle-stimulating hormone (FSH), androstenedione (A4) and sex hormone binding globulin (SHBG) were determined with chemiluminescent immunometric assays (Immulite XPi instrument, Siemens Healthcare Diagnostics, USA). Total testosterone (TT) was determined with electrochemiluminescence immunoassays (Cobas 8000 instrument, Roche Diagnostics, Germany). Due to a change in clinical practice in January 2021, method of determination of A4 and TT was upgraded from immunoassays to liquid chromatography with tandem mass spectrometry (LC-MS/MS). A4 and TT was measured with this method in 36 samples. A conversion factor of 1.67 was applied to the androstenedione determination in these samples to match output from both methods. No conversion was required on the testosterone determination. Free testosterone (FT) was calculated from TT using the Ross algorithm (18) and bioavailable testosterone (BT) was calculated using the matching algorithm to the Ross algorithm (19).

Serum estrone (E1), estradiol (E2), Anti-Müllerian hormone (AMH) and dehydroepiandrosterone sulfate (DHEAS) concentrations were determined in the Endocrine Laboratory of the Amsterdam UMC. E1, E2 and DHEAS concentrations were measured in serum using in-house developed LC-MS/MS as previously described (20, 21). AMH was measured using an electrochemiluminescence immunoassay (e601, a Cobas 6000 instrument, Roche Diagnostics, Germany).

### MASLD

Different MASLD parameters were investigated in this study. Ultrasonography of the liver was performed using the iU22m xMatrix ultrasound system (Philips Medical Systems) with a broad bandwidth C5–1 transducer. The presence of hepatic steatosis was determined on the ultrasound by pediatric radiologists. Transient elastography was performed with FibroScan, using M or XL-probes as indicated by the FibroScan. The controlled attenuation parameter (CAP) from FibroScan, a measurement for liver fat concentration, was utilized in this study. Mean CAP values were computed from at least 10 measurements and were considered valid if IQR/median was <20%, indicating sufficient consistency between measurements. FibroScan was available in a subgroup of 75 participants, as it had been available in our clinic from August 2018 onwards.

These different MASLD parameters enable evaluation of various stages of MASLD. Steatosis will be assessed with CAP and ultrasound and steatohepatitis will be evaluated with ALT concentrations.

### Statistics

Data are presented as median [25th-75th percentile] or percentages. Normality of data was assessed with histograms and QQ-plots, outliers were assessed using boxplots. Missing data were not imputed. Differences between girls and boys were assessed using unpaired tests (Mann-Whitney U test or Chi-square test as appropriate). Regression analyses were used to assess associations between hormone parameters (as independent variable) and MASLD parameters (as dependent variable). Due to sample size limitation, models were run for each hormone separately. Interactions between sex and the investigated hormone were assessed with an interaction term (sex*hormone).

Associations between ALT concentrations and hormone parameters were assessed in linear regression analyses. Model 1 was adjusted for BMI z-score, model 2 adjusted for BMI z-score and sex, and model 3 was adjusted for BMI z-score, sex and age. The interaction term sex*hormone was also evaluated in model 3. If non-significant, the term was omitted. If significant, models were run separately per sex. Age is only added in the model for ALT, as ALT concentrations vary with age (22).

Linear regression models were also utilized to assess associations between CAP values and hormone parameters. Model 1 was unadjusted, model 2 adjusted for BMI z-score and model 3 adjusted for BMI z-score and sex. Interaction between sex and hormone was also evaluated in model 3. If the term was non-significant, it was omitted. If significant, models were run for each sex separately.

Logistic regression analyses were performed to assess association between presence of steatosis and hormone parameters. Model 1 was unadjusted, model 2 adjusted for BMI z-score and model 3 adjusted for BMI z-score and sex. The interaction term sex*hormone was also evaluated in model 3. If non-significant, it was omitted. If significant, models were run separately per sex.

To account for multiple testing, a two-sided p ≤ 0.01 was considered statistically significant. To provide more structure in results, results with p ≤ 0.05 have also been indicated. Statistical analysis was performed with IBM SPSS Statistics 28.0 (Armonk, NY: IBM Corp, USA).

## Results

A total of 290 children with overweight and obesity (51% girls), with a median age of 11.5 years and median BMI z-score of 3.1 SDS were included in this study (**Table 1**). Based on IOTF criteria, 29.7% of children had overweight, 38.3% had obesity and 32.1% had severe obesity. Forty-eight percent of children were prepubertal, 30% were intrapubertal and 22% were postpubertal.

**Table 1:** Baseline characteristics, stratified by sex.

Characteristics	Total (n=209)	Girls (n=148)	Boys (n=142)	p-value
**Age (y)**	11.5 [9.1 - 14.0]	11.1 [9.0 - 14.3]	11.5 [9.3 - 13.4]	0.879
**Sex (%)**		51.0	49.0	n/a
**BMI z-score (SDS)**	3.1 [2.7 - 3.7]	2.9 [2.5 - 3.4]	3.3 [2.9 - 4.0]	<0.001*
**IOTF criteria (%)**				0.564
- Overweight	29.7	32.4	26.8	
- Obesity	38.3	37.2	39.4	
- Severe obesity	32.1	3.4	33.8	
**Puberty stage (%)**				<0.001*
- Pre-pubertal (G/M 1)	47.6	37.2	58.5	
- Intra-pubertal (G/M 2-3)	30	33.8	26.1	
- Post-pubertal (G/M 4-5)	22.4	29.1	15.5	
**LH (U/L)**	0.6 [0.1 - 2.5]	1.0 [0.2 - 3.5]	0.4 [0.1 - 1.9]	0.004*
**FSH (U/L)**	2.0 [1.0 - 4.1]	2.6 [1.4 - 5.1]	1.7 [0.7 - 2.5]	<0.001*
**E1 (pmol/L)**	40 [19 - 96.]	66 [24 - 143]	30 [17 - 58]	<0.001*
**E2 (pmol/L)**	20 [10 - 86]	59 [10 - 140]	10 [10 - 45]	<0.001*
**TT (nmol/L)**	0.4 [0.3 - 1.3]	0.4 [0.3 - 0.9]	0.5 [0.3 - 5.6]	0.008*
**FT (pmol/L)**	6.7 [4.1 - 23.3]	6.5 [4.3 - 16.5]	7.2 [3.7 - 140.1]	**0.048†**
**BioT (pmol/L)**	156 [95 - 555]	152 [100 - 387]	167 [87 - 3283]	**0.048†**
**A4 (nmol/L)**	4.1 [2.1 - 7.2]	4.6 [2.4 – 8.1]	3.7 [1.8 - 6.2]	0.004*
**DHEAS (umol/L)**	2.2 [1.2 - 4.0]	2.0 [1.1 - 3.7]	2.7 [1.3 - 4.5]	0.117
**AMH (ng/mL)**	4.8 [2.3 - 31.0]	2.4 [1.3 - 3.8]	31.5 [7.8 - 62.3]	<0.001*
**SHBG (nmol/L)**	34 [21 - 50]	33 [22 - 50]	35 [18 - 52]	0.948
**ALT (U/L)**	22 [18 - 29]	20 [16 - 26]	24 [20 - 34]	<0.001*
**Steatosis (%)**	12.1	8.8	15.5	0.080
**FibroScan (n)**	n=75	n=40	n=35	
**CAP (dB/m)**	229 [197 - 281]	214 [192 - 260]	250 [201 - 308]	0.087

Data presented as median [interquartile range, Q1-Q3] or percentages. *significant if p≤0.01, †significant if p≤0.05. All significant p-values are bold. Testing girls vs. boys with Mann-Whitney U test or ChiSquare test as appropriate.

IOTF, International Obesity Taskforce; G/M, genital/mammae; LH, luteinizing hormone; FSH, follicle stimulating hormone; E1, estrone; E2, estradiol; TT, total testosterone; FT, free testosterone; BioT, bioavailable testosterone; A4, androstenedione; DHEAS, dehydroepiandrosterone sulfate; AMH, Anti-Müllerian hormone; SHBG, sex hormone-binding globulin; ALT, alanine aminotransferase; CAP, controlled attenuation parameter.

When stratified by sex (**Table 1**), BMI z-score was significantly elevated in boys compared to girls (♂ 3.3 SDS [2.9-4.0] vs. ♀ 2.9 SDS [2.5-3.4]; p<0.001). Distribution of puberty groups differed significantly between boys and girls (prepubertal ♂ 58.5% vs ♀ 37.2%; intrapubertal ♂ 26.1% vs ♀ 33.8%; postpubertal ♂ 15.5% vs ♀ 29.1%; p<0.001). Stratification of baseline characteristics by puberty group can be found in **Supplementary Table 1**.

### Hormone parameters

TT, FT, BioT and AMH concentrations were significantly higher in boys, while LH, FSH, E1, E2 and A4 were significantly higher in girls (see details in **Table 1**). Age, distribution among IOTF categories, serum DHEAS and SHBG concentrations did not significantly differ between girls and boys.

### MASLD parameters

ALT concentrations were significantly higher in boys compared to girls (♂ 24 U/L [20 - 34] vs ♀ 20 [16 - 26]; p<0.001), while presence of steatosis and CAP values tended to be elevated in boys compared to girls (**Table 1**).

**Figure 1:**
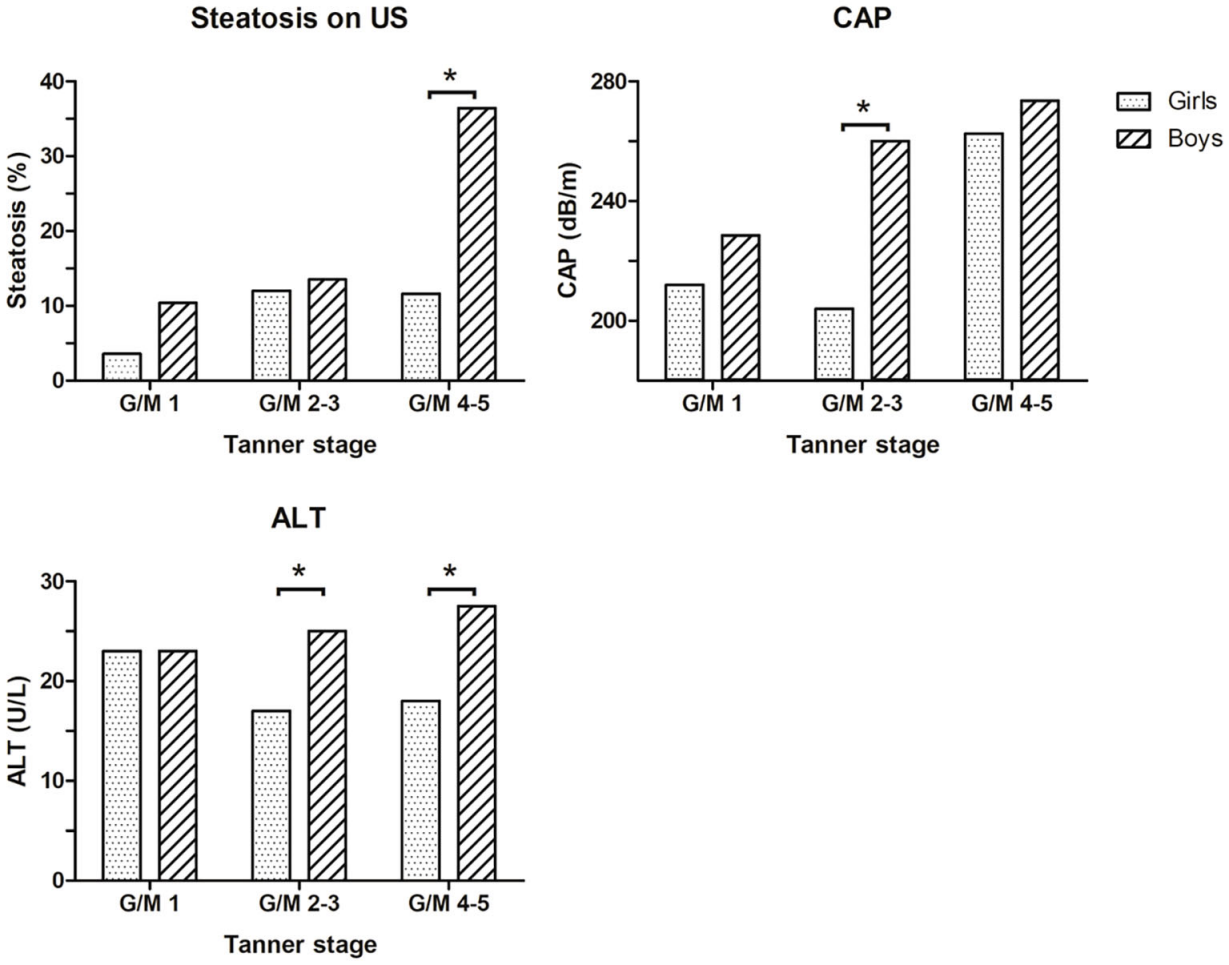
Steatosis on ultrasound, CAP and ALT per sex and G/M Tanner stage. *Statistically significant (p ≤ 0.05) when testing girls vs boys with Mann-Whitney U test or ChiSquare test as appropriate. ALT, alanine aminotransferase; CAP, controlled attenuation parameter; G/M, genital/mammae; US, ultrasound.

Prevalence of steatosis on ultrasound, ALT concentrations and CAP values are plotted per sex and different puberty group in **Figure 1**. Prevalence of steatosis was significantly elevated in postpubertal boys compared to girls (♂ 36.4% vs. ♀ 11.6%, p=0.018). CAP values were significantly elevated in intrapubertal boys compared to girls (♂ 260 dB/m vs. ♀ 204 dB/m, p=0.009). Intrapubertal and postpubertal boys presented with significantly elevated ALT concentrations compared to girls in respective groups (intrapubertal ♂ 25 U/L vs. ♀ 17 U/L, p<0.001; postpubertal ♂ 27 U/L vs ♀ 18 U/L, p<0.001).

### Associations between hormone parameters and ALT concentrations

Multiple linear regression analyses were performed to assess the associations between hormone parameters and ALT concentrations (**Table 2**). SHBG concentrations inversely associated with ALT concentrations, independent of BMI z-score, sex and age (-0.185 (-0.277 – -0.093), p<0.001). E2 and AMH showed an inverse trend association with ALT concentrations, when adjusting for BMI z-score, sex and age (E2 -0.027 (-0.049 – -0.005), p=0.018; AMH -0.096 (-0.192 – -0.001), p=0.048).

**Table 2:** Associations between hormone parameters and ALT.

Hormone parameters	Model 1		Model 2		Model 3	
	B (95% CI)	p-value	B (95% CI)	p-value	B (95% CI)	p-value
**LH (U/L)**	-0.143 (-0.446 – 0.161)	0.356	-0.059 (-0.364 – 0.245)	0.701	-0.207 (-0.530 – 0.116)	0.208
**FSH (U/L)**	0.190 (-0.637 – 1.017)	0.652	0.579 (-0.264 – 1.424)	0.177	0.129 (-0.849 – 1.107)	0.796
**E1 (pmol/L)**	-0.012 (-0.037 – 0.013)	0.348	0.004 (-0.023 – 0.030)	0.784	See table 3.	
**E2 (pmol/L)**	-0.016 (-0.033 – 0.002)	0.085	-0.007 (-0.025 – 0.012)	0.491	-0.027 (-0.049 – -0.005)	**0.018†**
**TT (nmol/L)**	0.737 (0.25 – 1.225)	0.003*	0.53 (0.011 – 1.048))	**0.045†**	0.331 (-0.257 – 0.918)	0.269
**FT (pmol/L)**	0.044 (0.021 – 0.066)	<0.001*	0.035 (-0.01 – 0.059)	0.006*	0.028 (-0.001 – 0.057)	0.057
**BioT (pmol/L)**	1.859 (0.881 – 2.836)	<0.001*	1.485 (0.436 – 2.535)	0.006*	1.199 (-0.035 – 2.433)	0.057
**A4 (nmol/L)**	0.071 (-0.104 – 0.246)	0.426	0.097 (-0.076 – 0.271)	0.269	See table 3.	
**DHEAS (umol/L)**	1.398 (0.588 – 2.209)	0.001*	1.271 (0.465 – 2.076)	0.002*	See table 3.	
**AMH (ng/mL)**	-0.010 (-0.082 – 0.062)	0.792	-0.122 (-0.209 – -0.034)	0.007*	-0.096 (-0.192 – -0.001)	**0.048†**
**SHBG (nmol/L)**	-0.186 (-0.268 – -0.103)	<0.001*	-0.188 (-0.269 – -0.107)	<0.001*	-0.185 (-0.277 – -0.093)	<0.001*

Linear regression analyses. B and 95% confidence interval are displayed, *significant if p≤0.01, †significant if p≤0.05. All significant p-values are bold.

Model 1: adjusted for BMI z-score. Model 2: adjusted for BMI z-score and sex. Model 3: adjusted for BMI z-score, sex and age.

Interaction term sex*hormone is evaluated in model 3. If not significant, it was omitted from model 3. If significant, model was run per sex separately (see **Table 3**). ALT, alanine aminotransferase; B, unstandardized regression coefficient; LH, luteinizing hormone; FSH, follicle stimulating hormone; E1, estrone; E2, estradiol; TT, total testosterone; FT, free testosterone; BioT, bioavailable testosterone; A4, androstenedione; DHEAS, dehydroepiandrosterone sulfate; AMH, Anti-Müllerian hormone; SHBG, sex hormone-binding globulin.

FT, BioT and DHEAS positively associated with ALT concentrations, when adjusted for BMI z-score and sex (FT 0.035 (-0.01 – 0.059), p=0.006; BioT 1.485 (0.436 – 2.535), p=0.006; DHEAS 1.271 (0.465 – 2.076), p=0.002). TT showed a positive trend association, independent of BMI z-score and sex (0.53 (0.011 – 1.048), p=0.045). LH and FSH concentrations were not significantly associated with ALT concentrations.

E1, A4, and DHEAS showed a significant interaction with sex in model 3 (p=0.002, p<0.001 and p<0.001 respectively), thus models were run per sex separately (see **Table 3**). Other hormones did not show interaction with sex.

**Table 3:** Associations between hormone parameters and ALT per sex.

Hormone parameters	Girls		Boys	
	B (95% CI)	p-value	B (95% CI)	p-value
**E1 (pmol/L)**	-0.016 (-0.049 - 0.018)	0.367	0.024 (-0.098 - 0.147)	0.695
**A4 (nmol/L)**	-0.004 (-0.135 - 0.128)	0.957	1.699 (0.528 - 2.870)	0.005*
**DHEAS (umol/L)**	0.132 (-1.044 - 1.308)	0.825	1.474 (-0.356 - 3.304)	0.114

Linear regression analyses. B and 95% confidence interval are displayed, *significant if p≤0.01, †significant if p≤0.05. All significant p-values are bold.

Models run per sex separately. Model adjusted for BMI z-score and age. B, unstandardized regression coefficient; E1, estrone; A4, androstenedione; DHEAS, dehydroepiandrosterone sulfate; SHBG, sex hormone-binding globulin.

Assessment per sex indicated that A4 was only significantly associated with ALT concentrations in boys, independent of BMI z-score and age (A4 ♂ 1.699 (0.528 - 2.870), p=0.005). E1 and DHEAS were not significantly associated with ALT concentrations in both girls and boys, when assessed per sex.

### Associations between hormone parameters and steatosis

Binominal logistic regression analyses were performed to assess associations between hormone parameters and presence of steatosis on ultrasound (**Table 4**). SHBG concentrations inversely associated with presence of steatosis, independent of BMI z-score and sex (0.948 (0.922 - 0.974), p<0.001). AMH showed an inverse trend association, when adjusting for BMI z-score and sex (0.98 (0.962 - 0.998), p=0.028). FT, BioT and DHEAS concentrations showed a positive trend association with presence of steatosis, when adjusted for BMI z-score (FT 1.004 (1.001 - 1.008), p=0.024; BioT 1.187 (1.023 - 1.377), p=0.024; DHEAS 1.155 (1.005 - 1.328), p=0.042). LH, FSH, E1, E2, TT and A4 concentrations showed no association with presence of steatosis.

**Table 4:** Associations between hormone parameters and steatosis.

Hormone parameters	Model 1		Model 2		Model 3	
	OR (95% CI)	p-value	OR (95% CI)	p-value	OR (95% CI)	p-value
**LH (U/L)**	0.994 (0.931 - 1.062)	0.863	0.997 (0.932 - 1.066)	0.934	1.006 (0.948 - 1.067)	0.846
**FSH (U/L)**	1.051 (0.91 - 1.213)	0.498	1.063 (0.921 - 1.227)	0.405	1.1 (0.949 - 1.276)	0.205
**E1 (pmol/L)**	1.001 (0.997 - 1.006)	0.530	1.001 (0.997 - 1.006)	0.537	1.003 (0.998 - 1.008)	0.178
**E2 (pmol/L)**	0.998 (0.994 - 1.002)	0.385	0.998 (0.994 - 1.003)	0.461	0.999 (0.995 - 1.004)	0.756
**TT (nmol/L)**	1.070 (0.995 - 1.150)	0.068	1.068 (0.993 - 1.149)	0.077	1.056 (0.977 - 1.141)	0.173
**FT (pmol/L)**	1.004 (1.001 - 1.008)	**0.019†**	1.004 (1.001 - 1.008)	**0.024†**	1.004 (1.000 - 1.007)	0.068
**BioT (pmol/L)**	1.193 (1.029 - 1.382)	**0.019†**	1.187 (1.023 - 1.377)	**0.024†**	1.165 (0.989 - 1.372)	0.068
**A4 (nmol/L)**	1.007 (0.984 - 1.031)	0.550	1.009 (0.986 - 1.033)	0.452	See text.	
**DHEAS (umol/L)**	1.152 (1.003 - 1.324)	**0.045†**	1.155 (1.005 - 1.328)	**0.042†**	1.146 (0.996 - 1.318)	0.057
**AMH (ng/mL)**	0.994 (0.98 - 1.009)	0.447	0.991 (0.976 - 1.006)	0.242	0.98 (0.962 - 0.998)	**0.028†**
**SHBG (nmol/L)**	0.946 (0.921 - 0.973)	<0.001*	0.949 (0.923 - 0.975)	<0.001*	0.948 (0.922 - 0.974)	<0.001*

Logistics regression analysis. Odds ratio and 95% confidence interval are displayed, *significant if p≤0.01, †significant if p≤0.05.

Model 1: unadjusted. Model 2: adjusted for and BMI z-score. Model 3: adjusted for BMI z-score and sex. Interaction term sex*hormone is evaluated in model 3. If not significant, it was omitted from model 3. If significant, the model was run per sex separately. OR, odd ratio; LH, luteinizing hormone; FSH, follicle stimulating hormone; E1, estrone; E2, estradiol; TT, total testosterone; FT, free testosterone; BioT, bioavailable testosterone; DHEAS, dehydroepiandrosterone sulfate; AMH, Anti-Müllerian hormone; SHBG, sex hormone-binding globulin.

A4 showed a significant interaction with sex in model 3 (p=0.008), thus the model was run separately per sex. Other hormones did not show interaction with sex. Assessment per sex indicated that A4 only positively associated with steatosis in boys, independent of BMI z-score (A4 ♂ 1.107 (1.042 - 1.313), p=0.008).

### Associations between hormone parameters and CAP values

Multiple linear regression analyses were performed to assess the associations between hormone parameters and CAP values measured with FibroScan (**Table 5**).

**Table 5:** Associations between hormone parameters and CAP.

Hormone parameters	Model 1		Model 2		Model 3	
	B (95% CI)	p-value	B (95% CI)	p-value	B (95% CI)	p-value
**LH (U/L)**	6.219 (0.197 - 12.241)	**0.043†**	4.768 (-1.160 - 10.695)	0.113	6.048 (-0.072 - 12.169)	0.053
**FSH (U/L)**	5.823 (-1.344 - 12.991)	0.110	5.897 (-0.932 - 12.725)	0.089	7.694 (0.584 - 14.803)	**0.034†**
**E1 (pmol/L)**	0.189 (-0.022 - 0.4)	0.078	0.117 (-0.095 - 0.328)	0.275	0.212 (-0.022 - 0.446)	0.075
**E2 (pmol/L)**	0.106 (-0.107 - 0.318)	0.092	0.100 (-0.103 - 0.303)	0.328	0.169 (-0.049 - 0.387)	0.128
**TT (nmol/L)**	5.864 (1.185 - 10.543)	**0.044†**	5.343 (0.885 - 9.831)	**0.020†**	5.227 (0.249 - 10.205)	**0.040†**
**FT (pmol/L)**	0.253 (0.049 - 0.458)	**0.016†**	0.217 (0.019 - 0.415)	**0.032†**	0.207 (-0.011 - 0.426)	0.062
**BioT (pmol/L)**	10.797 (2.07 - 19.523)	**0.016†**	9.727 (0.816 - 17.727)	**0.032†**	8.849 (-0.46 - 18.158)	0.062
**A4 (nmol/L)**	4.871 (1.223 - 8.519)	**0.010†**	4.094 (0.529 - 7.659)	**0.025†**	5.142 (1.47 - 8.813)	0.007*
**DHEAS (umol/L)**	11.546 (4.569 - 18.523)	0.002*	10.994 (4.342 - 17.647)	0.002*	8.328 (3.875 - 17.445)	0.003*
**AMH (ng/mL)**	-0.136 (-0.771 - 0.498)	0.670	-0.268 (-0.876 - 0.34)	0.383	-0.815 (-1.591 - -0.04)	**0.040†**
**SHBG (nmol/L)**	-1.080 (-1.610 - -0.549)	<0.001*	-0.960 (-1.483 - -0.437)	<0.001*	-0.937 (-1.466 - -0.407)	<0.001*

Linear regression analyses. B and 95% confidence interval are displayed, *significant if p≤0.01, †significant if p≤0.05. All significant p-values are bold.

Model 1: unadjusted. Model 2: adjusted for BMI z-score. Model 3: adjusted for BMI z-score and sex.

Interaction term sex*hormone is evaluated in model 3. If not significant, it was omitted from model 3. If significant, the model was run per sex separately. B, unstandardized regression coefficient; CAP, controlled attenuation parameter; LH, luteinizing hormone; FSH, follicle stimulating hormone; E1, estrone; E2: estradiol; TT, total testosterone; FT, free testosterone; BioT, bioavailable testosterone; A4, androstenedione; DHEAS, dehydroepiandrosterone sulfate; AMH, Anti-Müllerian hormone; SHBG, sex hormone-binding globulin.

A4 and DHEAS were positively associated with CAP, when adjusted for sex and BMI z-score (A4 5.142 (1.47 - 8.813), p=0.007; DHEAS 8.328 (3.875 - 17.445), p=0.003). FSH and TT showed a positive trend association with CAP, when adjusting for sex and BMI z-score (FSH 7.694 (0.584 - 14.803), p=0.034; TT 5.227 (0.249 - 10.205), p=0.04). SHBG was inversely associated with CAP values, independent of sex and BMI z-score (-0.937 (-1.466 – -0.407), p<0.001), while AMH showed an inverse trend (-0.815 (-1.591 – -0.04), p=0.04). FT and BioT showed a positive trend association with CAP, when adjusted for BMI z-score (FT 0.217 (0.019 - 0.415), p=0.032; BioT 9.727 (0.816 - 17.727). p=0.032).

## Discussion

This study investigated the association between a comprehensive set of sex hormones and MASLD in children with overweight and obesity. We found that estradiol, AMH and SHBG inversely associate with MASLD parameters, while androgens, including total, free and bioavailable testosterone, androstenedione and DHEAS, positively associate with MASLD parameters.

The negative association between estradiol concentration and ALT in children with overweight and obesity as found in this study, was independent of age, sex and BMI z-score. Elevated ALT concentrations are widely used in (pediatric) clinical practice as a marker for hepatocellular damage (23). According to the multiple hit theory (24), hepatic lipid accumulation in MASLD is associated with the generation of reactive oxidative species, a hallmark of oxidative stress. Excess reactive oxidative species formation can adversely disturb cellular metabolism and impact the functioning of organelles including mitochondria and the endoplasmic reticulum, leading to misfolding of newly synthesized proteins. Together with accumulation of lipotoxic lipid intermediates, this condition of metabolic stress is characterized by activation of c-Jun-N-terminal kinase (JNK) and nuclear factor kappa-light-chain-enhancer of activated B-cells (NF-κB) signaling pathways, which stimulate the production of pro-inflammatory cytokines, including interleukin-1β (IL-1β), interleukin-6 (IL-6) and tumor necrosis factor alpha (TNFα), by hepatocytes, Kupffer cells and hepatic stellate cells. The resulting pro-inflammatory response promotes recruitment of immune cells that intensify the inflammatory process. The associated hepatocellular damage (during which ALT can be released into the bloodstream), fosters further immune cell infiltration and hepatic inflammation, driving the progression from steatosis to steatohepatitis (8).

The aforementioned negative association between estradiol concentration and ALT in our study is corroborated in a recent pediatric study, which reports that higher estradiol concentrations are associated with lower grades of portal inflammation in children with biopsy-proven MASLD (11). Studies performed in pre-clinical settings also support the observed inverse relationship between estradiol and ALT concentrations in this study. They indicate that estradiol can limit MASLD progression by shifting macrophages from a pro-inflammatory to more anti-inflammatory phenotype, thus contributing to inflammation resolution (25, 26). Estrogens can also inhibit JNK and NF-κB signaling pathways, reducing the expression of genes encoding inflammatory mediators, including TNFα, IL-1β and IL-6 and thus preventing a chronic inflammatory status (27–30). Finally, studies using murine models have demonstrated that estrogens can limit liver damage by adapting cell proliferation and promoting hepatic regeneration (8).

Free, bioavailable testosterone and DHEAS associated significantly with ALT concentrations in this study, independent of BMI z-score and sex. No interaction with sex was present for these parameters. Preclinical and clinical adult studies have indicated that elevated androgen concentrations facilitate MASLD development and progression in females (8), while low testosterone levels have been associated with MASLD in adult males (8, 9). In a pediatric study, children with higher testosterone levels were less likely to have portal inflammation compared to lower testosterone levels, suggesting that testosterone may influence the spatial distribution of inflammation in the liver (11).

This study did not find associations between estradiol and hepatic steatosis parameters. Mueller et al. demonstrated similar findings in their pediatric cohort children with MASLD (11). This contrasts with adult studies that demonstrated that decreased estradiol concentrations are associated with MASLD in both men and women, while increased estradiol concentrations protect against development of MASLD in women. An explanation for the absent association between estradiol and steatosis parameters in our study and the study by Mueller et al. may lie in the broad range of estradiol concentrations found, as estradiol was not measured at a specific time point in the menstrual cycle. In addition to this, none of the children in this study population presented with aberrant concentrations of estradiol for their puberty stage, as opposed to several adult populations studied in literature that did present with altered concentrations of estradiol.

Free, bioavailable testosterone and DHEAS were positively associated with steatosis on ultrasound in children with overweight and obesity in this study, independent of BMI z-score. No interaction with sex was present for these parameters. Total testosterone and DHEAS were also associated with CAP values, independent from BMI z-score and sex, while free and bioavailable testosterone were associated with CAP values, independent of BMI z-score. Results from studies performed in (pre)clinical settings are in line with these findings, indicating that high concentrations of androgens trigger MASLD development in females via increased hepatic lipid storage and inducing insulin resistance (8, 9). Notably, results from a prospective population-based multicenter study showed that increasing free testosterone concentrations in women are associated with MASLD, independent of insulin resistance, BMI, waist circumference and serum lipid concentrations (31). Moreover, the association between free testosterone and MASLD persisted in women without androgen excess (31). High testosterone was also positively associated with steatosis severity in girls with biopsy confirmed MASLD, but not in boys (11).

In contrast, most studies in males indicate that decreased concentrations of androgens are associated with steatosis (8, 9), with testosterone treatment in hypo-androgenic populations leading to amelioration of MASLD (32, 33). However, rodent studies have indicated that the beneficial effect of testosterone treatment may partly be due to conversion of testosterone to estrogen (34–36). An explanation for not finding a similar association between low testosterone and steatosis in our study, may be that none of the boys presented with decreased concentrations of testosterone.

SHBG was significantly inversely associated with all MASLD parameters in this study. These findings are widely supported in literature. Mueller et al. reported that lower SHGB concentrations were inversely associated with steatosis severity in boys and girls with MASLD, but only with portal inflammation in girls with MASLD (11). Studies in men, postmenopausal women and women with PCOS also indicate an inverse association between SHBG and MASLD (8). Rodent studies have shown that overexpression of SHBG protects against high-fat-diet induced hepatic steatosis (37). SHBG may also limit MASLD progression by mitigating endoplasmic stress in hepatocyte cells (38).

The findings in this study underline the importance of considering sex differences in pediatric MASLD research and clinical practice. Future research could focus on sex-specific cut-off values for diagnostic tools, enabling earlier detection and treatment of MASLD. This may be especially relevant for boys, as previous research has shown that younger boys respond better to combined lifestyle intervention than older peers (39). Earlier treatment may also improve overall health outcomes for children with MASLD, as studies have shown that youth with MASLD present with increased hallmarks of cardiovascular disease, including significantly increased carotid intima-media thickness (a marker for subclinical atherosclerosis (40) and diastolic cardiac dysfunction (a major contributor to the development of health failure (41, 42). PCOS is also more prevalent in girls with MASLD, compared to girls without MASLD (43).

In addition to these clinical implications, the observed associations between sex hormones and MASLD may contribute to a better understanding of MASLD pathophysiology. The inverse association between estradiol and MASLD supports the hypothesis that estrogens may exert protective effects against hepatic inflammation from early age onwards. This may also help explain why MASLD prevalence is generally lower in premenopausal women and increases after menopause (4, 6). Improved understanding of these mechanisms could support the development of sex-specific prevention and future targeted therapies for MASLD.

### Strengths and limitations

This study is the first to investigate associations between a comprehensive set of sex hormones and different MASLD parameters in a European population. In addition to this, this study is the first to investigate associations between LH, FSH, free and bioavailable testosterone and MASLD parameters in a pediatric population. Furthermore, a majority of sex steroids were measured using LC-MS/MS, which is considered superior in sensitivity and specificity of measurements compared to currently available immunoassays (21). Lastly, different non-invasive MASLD parameters were utilized, enabling investigation of various stages of MASLD, including steatosis with CAP and ultrasound and steatohepatitis with ALT concentrations. A limitation of this study is that no information was available on whether blood sampling was performed during the follicular or luteal phase of the cycle of intra- and postpubertal girls, which may have led to increased variability in estradiol concentrations in this group. Another limitation of this study is that there were no liver biopsies available for participants. Although liver biopsy is considered the golden standard to diagnose steatohepatitis and fibrosis, it is sparingly utilized in pediatric practice due to potential associated complications in children.

## Conclusions

Estradiol, AMH and SHBG inversely associate with MASLD parameters, while androgens (total, free and bioavailable testosterone, androstenedione and DHEAS) positively associate with MASLD parameters (steatosis on ultrasound, CAP measured with FibroScan and ALT concentrations) in children with overweight, obesity and severe obesity, suggesting that sex hormones may play an important role in MASLD.

## Data Availability

The datasets presented in this article are not readily available because restrictions apply to the availability of the data generated and analyzed in this study as it pertains data of minors. The corresponding author will detail on request any restrictions and conditions under which access to deidentified data may be provided. Requests to access the datasets should be directed to a.vreugdenhil@mumc.nl.
