# Supplementary material for: Estradiol negatively associates with metabolic dysfunction-associated steatotic liver disease in children
Source: Front Endocrinol (Lausanne). 2026 Jul 6;17:1840658. doi: 10.3389/fendo.2026.1840658 (PMC13381234; doi:10.3389/fendo.2026.1840658)
Supplement: Supplementary file 1 [file Table1.pdf]

Supplementary Table 1. Baseline characteristics, stratified by puberty stage

	Total (n=290)	Pre-pubertal (n=138)	Intra-pubertal (n=87)	Post-pubertal (n=65)	p-value
**Age (y)**	11.5 [9.1-14.0]	9.2 [7.6-11.0]	12.1 [20.9-13.3]	15.8 [14.4-16.7]	**<0.001***
**Sex (% girls)**		39.9	57.5	66.2	**<0.001***
**BMI z-score (SDS)**	3.1 [2.7-3.7]	3.1 [2.7-3.8]	3.1 [2.7-3.6]	3.2 [2.6-3.7]	0.608
**IOTF criteria (%)**					
- Overweight	29.7	30.4	29.9	27.7	0.922
- Obesity	38.3	36.2	41.1	38.5	
- Severe obesity	32.1	33.3	28.7	33.8	
**ALT (U/L)**	22 [18-29]	23 [19-29]	21 [16-29]	20 [16-33]	0.085
**LH (U/L)**	0.6 [0.1-2.5]	0.1 [0.1-0.25]	1.4 [0.4-2.7]	3.1 [2.3-6.6]	**<0.001***
**FSH (U/L)**	2.0 [1.0-4.1]	01.2 [0.7-1.9]	3.4 [1.8-5.3]	3.9 [2.2-5.3]	**<0.001***
**E1 (pmol/L)**	40 [19-96]	19 [12-30]	57 [36-104]	127 [81-212]	**<0.001***
**E2 (pmol/L)**	20 [10-85]	10 [10-10]	44 [18-100]	117 [73-201]	**<0.001***
**TT (nmol/L)**	0.4 [0.3-1.3]	0.3 [0.3-0.3]	0.7 [0.3-2.5]	1.5 [1.0-8.2]	**<0.001***
**FT (pmol/L)**	6.7 [4.1-23.3]	4.3 [3.2-5.7]	10.9 [5.4-39.7]	29.2 [16.7-211.0]	**<0.001***
**BioT (pmol/L)**	156 [95-555]	100 [76-135]	255 [128-929]	684 [391-4945]	**<0.001***
**A4 (nmol/L)**	4.1 [2.1-7.2]	2.3 [1.4 – 4.0]	4.7 [3.0 – 6.9]	9.9 [6.4 – 11.6]	**<0.001***
**DHEAS (umol/L)**	2.2 [1.2-4.0]	1.3 [0.7-2.2]	2.9 [1.6-3.7]	5.1 [3.6-7.0]	**<0.001***
**AMH (ng/mL)**	4.8 [2.3-31.0]	23.3 [2.5-61.6]	4.6 [1.82-7.99]	3.4 [2.5-5.3]	**<0.001***
**SHBG (nmol/L)**	34 [21-50]	43 [30-58]	29 [18-39]	22 [15-34]	**<0.001***
**Steatosis (%)**	12.1	8.0	12.6	20.0	0.082
**FibroScan**	n=75	n=31	n=30	n=14	
**CAP (dB/m)**	229 [197-281]	226 [185-262]	219 [197-278]	270 [213-309]	0.172

Data presented as median [IQR] or percentages. *significant if p≤0.01, †significant if p≤0.05. All significant p-values are bold. Testing puberty stages against one another with Kruskall-Wallis tests or ChiSquare tests as appropriate. Abbreviations: IOTF: International Obesity Taskforce; G/M: genital/mammae; LH: luteinizing hormone; FSH: follicle stimulating hormone; E1: estrone; E2: estradiol; TT: total testosterone; FT: free testosterone; BioT: bioavailable testosterone; A4: androstenedione; DHEAS: dehydroepiandrosterone sulfate; AMH: Anti-Müllerian hormone; SHBG: sex hormone-binding globulin; ALT: alanine aminotransferase; CAP: controlled attenuation parameter.
